# Mechanism of differential sensitivity of human bladder cancer cells to mitomycin C and its analogue.

**DOI:** 10.1038/bjc.1994.46

**Published:** 1994-02

**Authors:** B. H. Xu, V. Gupta, S. V. Singh

**Affiliations:** Cancer Research Laboratory, Mercy Cancer Center, Mercy Hospital, Pittsburgh, Pennsylvania 15219.

## Abstract

This study was undertaken to elucidate the mechanism(s) of differential sensitivity of human bladder cancer cell lines J82 and SCaBER to mitomycin C (MMC) and its analogue, BMY 25067. The IC50 values for MMC and BMY 25067 in the SCaBER cell line were respectively 5- and 4-fold higher than in J82. BMY 25282 and BMY 25067 were significantly more cytotoxic, on a molar basis, than MMC in both the cell lines. NADPH cytochrome P450 reductase and DT diaphorase activities were significantly higher in the J82 cell line than in SCaBER, suggesting that relatively lower sensitivity of the SCaBER cell line to MMC and BMY 25067 may be due to deficient drug activation. This conclusion was supported by the observation that IC50 values for BMY 25282, which has lower quinone reduction potential than MMC and BMY 25067, did not differ significantly in these cell lines. A correlation between drug sensitivity, oxyradical formation and levels of antioxidative enzymes was not observed. These results suggest that the relatively lower sensitivity of SCaBER cells to MMC or BMY 25067 may be independent of differential oxyradical formation. MMC-induced DNA interstrand cross-link (ISC) formation was markedly lower in the SCaBER cell line than in J82. However, it remains to be seen if the reduced ISC frequency in the SCaBER cell line is a consequence of deficient drug activation or results from increased repair of the damaged DNA.


Br. J. Cancer (1994), 69, 242–246 © Macmillan Press Ltd., 1994

### Mechanism of differential sensitivity of human bladder cancer cells to mitomycin C and its analogue

B.H. Xu, V. Gupta & S.V. Singh

*Cancer Research Laboratory, Mercy Cancer Center, The Mercy Hospital, Pittsburgh, Pennsylvania 15219, USA.*

**Summary** This study was undertaken to elucidate the mechanism(s) of differential sensitivity of human bladder cancer cell lines J82 and SCaBER to mitomycin C (MMC) and its analogue, BMY 25067. The $IC_{50}$ values for MMC and BMY 25067 in the SCaBER cell line were respectively 5- and 4-fold higher than in J82. BMY 25282 and BMY 25067 were significantly more cytotoxic, on a molar basis, than MMC in both the cell lines. NADPH cytochrome P450 reductase and DT diaphorase activities were significantly higher in the J82 cell line than in SCaBER, suggesting that relatively lower sensitivity of the SCaBER cell line to MMC and BMY 25067 may be due to deficient drug activation. This conclusion was supported by the observation that $IC_{50}$ values for BMY 25282, which has lower quinone reduction potential than MMC and BMY 25067, did not differ significantly in these cell lines. A correlation between drug sensitivity, oxyradical formation and levels of antioxidative enzymes was not observed. These results suggest that the relatively lower sensitivity of SCaBER cells to MMC or BMY 25067 may be independent of differential oxyradical formation. MMC-induced DNA interstrand cross-link (ISC) formation was markedly lower in the SCaBER cell line than in J82. However, it remains to be seen if the reduced ISC frequency in the SCaBER cell line is a consequence of deficient drug activation or results from increased repair of the damaged DNA.

Mitomycin C (MMC) is widely used in the treatment of various solid tumours, including human bladder carcinoma (Crooke & Bradner, 1976; Carter, 1979). However, the frequent occurrence of resistance to MMC limits its clinical effectiveness (Moertel *et al.*, 1968; Lenaz, 1985). Elucidation of the biochemical mechanism(s) leading to MMC resistance may, therefore, be essential for developing strategies to increase the therapeutic value of this drug.

MMC requires enzymatic bioactivation, and metabolic pathways for one- and two-electron reduction of MMC leading to the generation of cytotoxic species have been described (Iyer & Szybalski, 1963; Pan *et al.*, 1984; Tomasz *et al.*, 1987; Siegel *et al.*, 1992). While many enzymes have been implicated in MMC bioactivation, NADPH cytochrome P450 reductase and DT diaphorase appear to be the key enzymes involved in its bioreductive activation (Keyes *et al.*, 1984; Pan *et al.*, 1984; Hoban *et al.*, 1990; Gustafson & Pritsos, 1992; Siegel *et al.*, 1992). Whereas the most abundant lesion produced by enzymatically activated MMC is the DNA monoadduct, MMC is believed to exert cytotoxic activity primarily through the formation of DNA–DNA cross-links (Long *et al.*, 1984; Dorr *et al.*, 1985; Tomasz *et al.*, 1987). MMC-dependent oxyradicals have also been proposed to contribute to its cytotoxicity (Pan *et al.*, 1984; Pritsos & Sartorelli, 1986; Dusre *et al.*, 1990).

Several independent investigators have shown that cellular resistance to MMC often results from deficient drug activation due to the down-regulation of bioactivation enzymes (Willson *et al.*, 1985, 1987; Marshall *et al.*, 1989; Hoban *et al.*, 1990; Pan *et al.*, 1992). On the other hand, MMC resistance in a subclone of the L1210 leukaemic cell line appears to be due to reduced drug accumulation (Dorr *et al.*, 1987). Cross-resistance to MMC in a multidrug-resistant clone of the MCF-7 human breast cancer cell line (ADR), selected for resistance to doxorubicin, has been attributed to the reduced formation of MMC-dependent oxyradicals (Dusre *et al.*, 1990).

Recent studies suggest that GSH/GST-mediated drug inactivation may also play an important role in MMC resistance (Perry *et al.*, 1992; Siegel *et al.*, 1992; Singh *et al.*, 1992; Xu & Singh, 1992*a*). We have shown that cross-resistance to MMC in a multidrug-resistant variant of P388 mouse leukaemia (P388/R-84) is independent of either deficient drug activation or reduced drug accumulation, but seems to be influenced by GSH/GST levels (Singh *et al.*, 1992; Xu & Singh, 1992*a*,*b*). Subsequently, using a panel of three unrelated human bladder cancer cell lines (J82, HT-1197 and SCaBER) differing in their sensitivities to MMC, we noticed a good correlation between MMC cytotoxicity and GST level, activity being highest in the relatively insensitive cell line (SCaBER) and lowest in the comparatively sensitive cell line, J82 (Xu *et al.*, 1993). In addition, the results from this study suggested that multiple mechanisms may be responsible for the relatively lower sensitivity of SCaBER cells to MMC. We have extended the study and compared other mechanisms of MMC resistance, including the levels of bioactivation enzymes, oxyradical formation, antioxidative enzyme levels and MMC-induced DNA cross-link formation in J82 and SCaBER cell lines, which are sensitive and relatively insensitive, respectively, to MMC and its analogue, BMY 25067.

### Materials and methods

### *Chemicals*

MMC and its analogues were generously supplied by the Bristol Myers Squibb Company. Sources of other chemicals were as described previously (Xu & Singh, 1992*a*, *b*). Stock solution of MMC was prepared in phosphate-buffered saline (PBS), whereas MMC analogues BMY 25282 and BMY 25067 were dissolved in dimethylsulphoxide (DMSO). Fresh drug solutions were prepared immediately before use. The final concentration of DMSO was $\leqslant 0.5\%$, which did not affect the colony-forming ability of either of the cell lines.

### *Cell lines*

Human bladder cancer cells, J82 and SCaBER, were obtained from the American Type Culture Collection (Rockville, MD, USA). Monolayer cultures were maintained in Eagle's minimum essential medium supplemented with non-essential amino acids, sodium pyruvate, 10% fetal bovine serum and antibiotics. The plating efficiencies of J82 and SCaBER cells were $27 \pm 5\%$ and $45 \pm 13\%$ ($n = 9$) respectively. The cell doubling times for J82 and SCaBER cell lines were similar.

Correspondence: S.V. Singh.
Received 2 June 1993; and in revised form 27 September 1993.

*Cell survival assay*

The *in vitro* cytotoxicity of MMC and its analogues in human bladder cancer cell lines was determined by colony formation assay. Briefly, $3 \times 10^3$ cells were allowed to attach overnight. The cells were exposed to different concentrations of the drug for 1 h at 37°C. After washing the cells twice with PBS, fresh medium was added and the flasks were incubated for 8–10 days at 37°C in an atmosphere of 5% carbon dioxide and 95% air. Colonies containing more than 50 cells were counted under an inverted microscope. The $IC_{50}$ value (drug concentration producing 50% cell growth inhibition) was determined by plotting percentage cell survival *vs* drug concentration. Statistical significance was determined by Student's *t*-test.

*Enzyme assays*

NADPH cytochrome P450 reductase activity was determined by the procedure described by Hrycay *et al.* (1975). DT diaphorase was measured according to the method described by Ernster (1967) using 2,6-dichlorophenol indophenol (DCPIP) as a substrate. The concentration of dicoumarol used to study the inhibition of the reduction of DCPIP was 10 μM. GSH peroxidase and catalase activities were determined by the methods described by Beutler (1984). Protein content was determined by the method of Bradford (1976).

*MMC-dependent lipid peroxidation*

MMC-dependent oxyradical formation in J82 and SCaBER cell lines was compared by monitoring the production of 2-thiobarbituric acid-reactive malondialdehyde (an indicator of oxyradical formation). Malondialdehyde content was determined according to the method described by Konings & Drijver (1979) with slight modifications. The details of the lipid peroxidation assay have been described by us previously (Xu & Singh, 1992*b*).

*Alkaline elution assay*

Cells ($0.5 \times 10^6$ in 5 ml of complete medium) were labelled with [$^{14}$C]thymidine (0.02 μCi ml$^{-1}$; specific activity, 56 mCi mmol$^{-1}$) for 48 h. The medium was removed and the cells were washed twice with PBS. The radioactivity was chased by a 24 h post-incubation at 37°C in fresh medium containing 10 μM non-radioactive thymidine. The labelled cells were incubated with different concentrations of MMC for 1 h at 37°C, and washed twice with ice-cold PBS. Aliquots containing approximately $1 \times 10^6$ cells were irradiated with 15 Gy of γ-radiation on ice. DNA cross-link formation was determined by using the alkaline elution technique as described by Kohn *et al.* (1981). Interstrand cross-link (ISC) frequency was calculated by using the equation:

$$\text{ISC (Gy eq.)} = \{[(1\text{-}R_0)/(1\text{-}R_1)]^{\frac{1}{2}}\text{-}1\} \times 15$$

where $R_0$ and $R_1$ represent the fraction of DNA retained from control and MMC-treated cells respectively.

### Results

The *in vitro* cytotoxicity of MMC, BMY 25282 and BMY 25067 in J82 and SCaBER cell lines is shown in Figure 1. The $IC_{50}$ value for MMC in SCaBER cells (1.5 ± 0.15 μM) was 5-fold higher ($P<0.001$) than in J82 (0.3 ± 0.04 μM) (Figure 1a). BMY 25282 and BMY 25067 appeared to be significantly more cytotoxic, on a molar basis, than the parent drug in both these cell lines (Figure 1b and c). The $IC_{50}$ values for BMY 25282 in J82 and SCaBER cells were 0.026 ± 0.005 and 0.039 ± 0.012 μM, respectively, which did not differ significantly ($P = 0.14$). Thus, BMY 25282 was approximately 12- and 38-fold more cytotoxic than the parent drug in J82 and SCaBER cells respectively. BMY 25067 was 10- and 12.5-fold more active than MMC in J82 and SCaBER cells respectively. The $IC_{50}$ values for BMY 25067 in J82 and SCaBER cells were 0.03 ± 0.001 and 0.12 ± 0.006 μM respectively. The SCaBER cells displayed a 4-fold higher $IC_{50}$ value for BMY 25067 ($P<0.001$) compared with the J82 cells.

**Figure 1** Survival of cells exposed to various concentrations of **a**, MMC; **b**, BMY 25282; and **c**, BMY 25067: (○) J82; (●) SCaBER. Points represent mean ± s.d. of three independent experiments.

Table I shows the levels of key MMC bioactivation enzymes in these cell lines. NADPH cytochrome P450 reductase activity in SCaBER cells was about 31% of that in the J82 cell line. DT diaphorase activity was also significantly lower in SCaBER cells than in J82.

Table II compares MMC-dependent lipid peroxidation (an indicator of oxyradical formation) and the levels of cellular antioxidative enzymes in these cells. MMC-dependent lipid peroxidation was about 54% higher in the SCaBER cell line than in J82 ($P<0.005$). Interestingly, the antioxidative enzyme levels did not differ significantly between J82 and SCaBER cell lines (Table II). We have shown previously that the GSH level is also similar in J82 and SCaBER cell lines (Xu *et al.*, 1993).

Since cross-linking of DNA is believed to be important in the cytotoxic activity of MMC (Long *et al.*, 1984; Dorr *et al.*, 1985), ISC formation was compared in these cell lines (Figure

**Table I** NADPH cytochrome P450 reductase and DT diaphorase activities in 14,000 g supernatant fractions of J82 and SCaBER cells

| Cell line | NADPH cytochrome reductase (nmol min$^{-1}$ mg$^{-1}$ protein) | DT diaphorase (μmol min$^{-1}$ mg$^{-1}$ protein) |
|---|---|---|
| J82 | 26 ± 5[a] | 6 ± 2 |
| SCaBER | 8 ± 0.5[b] | 0.5 ± 0.02[c] |

[a]Values represent mean ± s.d. of three determinations. [b]Significantly different from J82, $P < 0.005$, by t-test. [c]Significantly different from J82, $P < 0.05$, by t-test.

**Table II** MMC-dependent lipid peroxidation and GSH peroxidase and catalase activities in J82 and SCaBER cells

| | Cell lines | |
|---|---|---|
| | J82 | SCaBER |
| Lipid peroxidation (nmol h$^{-1}$ mg$^{-1}$ protein) | 1.1 ± 0.05[a] | 1.7 ± 0.2[b] |
| GSH peroxidase activity[c] (nmol min$^{-1}$ mg$^{-1}$ protein) | 654 ± 170 | 435 ± 110 |
| Catalase activity[d] (μmol min$^{-1}$ mg$^{-1}$ protein) | 3 ± 0.1 | 3 ± 0.4 |

[a]Values represent mean ± s.d. of three determinations. [b]Significantly different from J82, $P < 0.005$, by t-test. [c]GSH peroxidase activity was measured using cumene hydroperoxide as a substrate. [d]Catalase activity was measured by monitoring the decomposition of hydrogen peroxide.

**Figure 2** DNA interstrand cross-links induced by different concentrations of MMC in J82 (○) and SCaBER (●) cell lines. Each point represents mean ± s.d. of two independent elution experiments.

2). MMC-induced ISC frequencies, at three drug concentrations, were markedly lower in the SCaBER cell line than in J82. The ISC frequencies induced by 0.75, 3.75 and 7.50 μM MMC in the SCaBER cell line were about 44%, 15% and 58%, respectively of those in J82 cells.

### Discussion

Whereas several different mechanisms have been proposed to account for MMC resistance (Dorr et al., 1987; Willson et al., 1987; Marshall et al., 1989; Dusre et al., 1990; Singh et al., 1992), deficient bioactivation of the drug appears to be the most frequently encountered mechanism (Willson et al., 1985; Hoban et al., 1990; Pan et al., 1992). This has led to the synthesis of MMC analogues, such as BMY 25282, which have much lower quinone reduction potential than the parent drug (Doyle & Vyas, 1990). Thus, BMY 25282 has exhibited superior anti-tumour activity in certain MMC-resistant cells (for review see Doyle & Vyas, 1990). In the present study also BMY 25282 appeared to be more active than MMC in both the cell lines. These results suggest that BMY 25282 may be a superior anti-tumour agent to MMC. Unfortunately, the toxicity of BMY 25282 has limited its clinical use (Doyle & Vyas, 1990).

While the reduction potentials of BMY 25067 and MMC are similar, this analogue has displayed much lower haematological toxicity such as neutropenia than MMC in preclinical studies (Bradner et al., 1990). Interestingly, both the bladder cell lines examined in this study were significantly more sensitive to BMY 25067 than MMC. Relatively higher cytotoxicity of BMY 25067 compared with MMC has also been reported in other cell lines (Dusre et al., 1990; Xu & Singh, 1992a). Similarly, BMY 25067 exhibited superior activity against B16 melanoma with a high percentage of cures in mice when both the tumours and drugs were given i.p. or in B16 melanoma implanted s.c. and BMY 25067 administered i.v. (Bradner et al., 1990). These results suggest that BMY 25067 may be seriously considered for further clinical development because of its superior cytotoxicity and lower toxicity than the parent compound.

The cytotoxicity of MMC is suggested to be mediated by DNA–DNA cross-linking and oxygen radical-mediated damage (Dorr et al., 1985; Pritsos & Sartorelli, 1986; Dusre et al., 1990). Although a good correlation has been reported between MMC cytotoxicity and DNA cross-link formation (Long et al., 1984; Dorr et al., 1985), the contribution of oxyradicals in the activity of this agent is not as clear. Dusre et al. (1990) have suggested that relatively lower sensitivity of the MCF-7.ADR cell line to MMC, BMY 25282 and BMY 25067 compared with wild-type MCF-7 cells results from the reduced oxygen radical formation. Previous studies from our laboratory (Xu & Singh, 1992b) have also shown reduced MMC-dependent oxyradical formation in P388/R-84 cells, which are significantly cross-resistant to MMC, compared with P388/S cells. Pritsos et al. (1986) suggested that, although oxygen radicals do contribute to the aerobic cytotoxicity of MMC and BMY 25282, the cytotoxic lesions occur at site(s) other than DNA. However, McGurl and Kennedy (1989) have concluded that oxyradicals may not play a role in the anti-tumour activity of this drug. In this study, sensitivity to MMC did not seem to correlate with oxyradical formation. In fact, MMC-dependent lipid peroxidation was significantly higher in MMC-resistant SCaBER cells. Although the present study does not provide evidence either for or against a role of oxyradicals in MMC cytotoxicity, this mechanism does not appear to contribute to the differential sensitivity of J82 and SCaBER cells to MMC.

In the present study, the activities of two key MMC bioactivation enzymes, NADPH cytochrome P450 reductase and DT diaphorase, were significantly lower in SCaBER cells than in J82 cells. These results suggest that the relatively lower sensitivity of the SCaBER cell line to MMC and perhaps BMY 25067 may be due to the deficient drug activation. The observation that the $IC_{50}$ values for BMY 25282 in J82 and SCaBER cell lines did not differ significantly provides further support for this premise.

MMC has been shown to be preferentially cytotoxic to hypoxic tumour cells (Kennedy et al., 1980; Rockwell, 1983).

Several MMC-resistant cell lines, with deficient drug activation as mechanism of MMC resistance, have exhibited parental sensitivity to this agent under hypoxic conditions (Dulhanty *et al.*, 1989; Marshall *et al.*, 1989; Hoban *et al.*, 1990). Although the experiments described in the present study were performed under aerobic conditions, it remains to be seen if the sensitivity of the SCaBER cell line to MMC is similar to that of J82 under hypoxic conditions.

The results of the present study suggest that, in addition to the deficient bioactivation, reduced DNA cross-linking may also contribute to the relatively lower sensitivity of SCaBER cells to MMC. However, further studies are needed to determine whether reduced DNA cross-linking in the SCaBER cell line is a consequence of deficient drug activation or results from the increased repair of the damaged DNA in these cells.

This investigation was supported by USPHS Grant CA 50638, awarded by the National Cancer Institute. The authors wish to thank Mr Mekala Sreevardhan for technical assistance.

**Abbreviations:** BMY 25282, 7-*N*-(dimethylaminomethylene)-mitomycin C; BMY 25067, *N*-7-[2-(4-nitrophenyldithio)ethyl]-mitomycin C; DCPIP, 2,6-dichlorophenol indophenol; GSH, glutathione: GST, glutathione transferase; $IC_{50}$, 50% inhibitory concentration; ISC, interstrand cross-link; MMC, mitomycin C; PBS, phosphate-buffered saline.

### References

BEUTLER, E. (1984). Glutathione peroxidase, catalase. In *Red Cell Metabolism: A Manual of Biochemical Methods*, Beutler, E. (ed.), pp. 74–76, 105–106. Grune & Stratton: New York.

BRADFORD, M.M. (1976). A rapid and sensitive method for the quantitation of microgram quantities of protein utilizing the principle of protein-dye binding. *Anal. Biochem.*, **72**, 248–254.

BRADNER, W.T., ROSE, W.C., SCHURIG, J.E. & FLORCZYK, A.P. (1990). Antitumour activity and toxicity in animals of *N*-7[2-(4-nitrophenyldithio)ethyl] mitomycin C (BMY-25067). *Invest. New Drugs*, **8** (Suppl. 1), S1–S7.

CARTER, S.K. (1979). Reflections and prospects. In *Mitomycin C, Current Status and New Developments*, Carter, S.K. & Crooke, S.T., (eds), pp. 251–254. Academic Press: New York.

CROOKE, S.T. & BRADNER, W.T. (1976). Mitomycin C: a review. *Cancer Treat. Rev.*, **3**, 121–139.

DORR, R.T., BOWDEN, G.T., ALBERTS, D.S. & LIDDIL, J.D. (1985). Interactions of mitomycin C with mammalian DNA detected by alkaline elution. *Cancer Res.*, **45**, 3510–3516.

DORR, R.T., LIDDIL, J.D., TRENT, J.M. & DALTON, W.S. (1987). Mitomycin C resistant L1210 leukemia cells: association with pleiotropic drug resistance. *Biochem. Pharmacol.*, **36**, 3115–3120.

DOYLE, T.W. & VYAS, D.M. (1990). Second generation analogs of etoposide and mitomycin C. *Cancer Treat. Rev.*, **17**, 127–131.

DULHANTY, A., LI, M. & WHITMORE, G.F. (1989). Isolation of chinese hamster ovary cell mutants deficient in excision repair and mitomycin C bioactivation. *Cancer Res.*, **49**, 117–122.

DUSRE, L., RAJAGOPALAN, S., ELIOT, H.M., COVEY, J.M. & SINHA, B.K. (1990). DNA interstrand cross-link and free radical formation in a human multidrug-resistant cell line from mitomycin C and its analogues. *Cancer Res.*, **50**, 648–652.

ERNSTER, L. (1967). DT diaphorase. *Methods Enzymol.*, **10**, 309–317.

GUSTAFSON, D.L. & PRITSOS, C.A. (1992). Bioactivation of mitomycin C by xanthine dehydrogenase from EMT6 mouse mammary carcinoma tumors. *J. Natl Cancer Inst.*, **84**, 1180–1185.

HOBAN, P.R., WALTON, M.I., ROBSON, C.N., GODDEN, J., STRATFORD, I.J., WORKMAN, P., HARRIS, A.L. & HICKSON, I.D. (1990). Decreased NADPH: cytochrome P-450 reductase activity and impaired drug activation in a mammalian cell line resistant to mitomycin C under aerobic but not hypoxic conditions. *Cancer Res.*, **50**, 4692–4697.

HRYCAY, E.G., JONEN, H.G., LU, A.Y.H. & LEVIN, W. (1975). Reconstitution of the reduced nicotinamide adenine dinucleotide phosphate- and reduced nicotinamide adenine dinucleotide-peroxidase activities from solubilized components of rat liver microsomes. *Arch. Biochem. Biophys.*, **166**, 145–151.

IYER, V.N. & SZYBALSKI, W. (1963). Mitomycin and porfiromycin: chemical mechanism of activation and cross-linking of DNA. *Microbiology*, **50**, 355–362.

KENNEDY, K.A., ROCKWELL, S. & SARTORELLI, A.C. (1980). Preferential activation of mitomycin C to cytotoxic metabolites by hypoxic tumor cells. *Cancer Res.*, **40**, 2356–2360.

KEYES, S.R., FRACASSO, P.M., HEIMBROOK, D.C., ROCKWELL, S., SLIGAR, S.G. & SARTORELLI, A.C. (1984). Role of NADPH: cytochrome *c* reductase and DT-diaphorase in the biotransformation of mitomycin C. *Cancer Res.*, **44**, 5638–5643.

KOHN, K.W., EWIG, R.A., ERICKSON, L.C. & ZWELLING, L.A. (1981). Measurements of strand-breaks and cross-links in DNA by alkaline elution. In *DNA Repair: A Laboratory Manual of Research Techniques*, Fredberg, E.C. & Hanawalt, P.C. (eds), pp. 379–401. Marcel Dekker: New York.

KONINGS, A.W.T. & DRIJVER, E.B. (1979). Radiation effects on membranes. I. Vitamin E deficiency and lipid peroxidation. *Radiat. Res.*, **80**, 494–501.

LENAZ, L. (1985). Mitomycin C in advanced breast cancer. *Cancer Treat. Rev.*, **12**, 235–249.

LONG, B.H., WILLSON, J.K.V., BRATTAIN, D.E., MUSIAL, S. & BRATTAIN, M.G. (1984). Effects of mitomycin on human colon carcinoma cells. *J. Natl Cancer Inst.*, **73**, 787–792.

MARSHALL, R.S., PATERSON, M.C. & RAUTH, A.M. (1989). Deficient activation by a human cell strain leads to mitomycin resistance under aerobic but not hypoxic conditions. *Br. J. Cancer*, **59**, 341–346.

MCGURL, J. & KENNEDY, K.A. (1989). Effect of glutathione (GSH) depletion by dimethylmaleate (DEM) or buthionine *S,R* sulfoximine (BSO) on mitomycin C (MC) toxicity towards EMT6 mouse mammary tumor cells. *Proc. Am. Assoc. Cancer Res.*, **30**, 543.

MOERTEL, C.G., REITEMEIER, R.J. & HAHN, R.G. (1968). Mitomycin C therapy in advanced gastrointestinal cancer. *J. Am. Med. Assoc.*, **204**, 1045–1048.

PAN, S., ANDREWS, P.A., GLOVER, C.J. & BACHUR, N.R. (1984). Reductive activation of mitomycin C and mitomycin C metabolites catalyzed by NADPH cytochrome P-450 reductase and xanthine oxidase. *J. Biol. Chem.*, **259**, 959–966.

PAN, S., AKMAN, S.A., FORREST, G.L., HIPSHER, C. & JOHNSON, R. (1992). The role of NAD(P)H:quinone oxidoreductase in mitomycin C-and porfiromycin-resistant HCT 116 human colon-cancer cells. *Cancer Chemother. Pharmacol.*, **31**, 23–31.

PERRY, R.R., GREAVES, B.R., RASBERRY, U. & BARRANCO, S.C. (1992). Effect of treatment duration and glutathione depletion on mitomycin C cytotoxicity *in vitro*. *Cancer Res.*, **52**, 4608–4612.

PRITSOS, C.A. & SARTORELLI, A.C. (1986). Generation of reactive oxygen radicals through bioactivation of mitomycin antibiotics. *Cancer Res.*, **46**, 3528–3532.

PRITSOS, C.A., KEYES, S.R. & SARTORELLI, A.C. (1986). Role of oxygen radicals in the cytotoxicity of mitomycin antibiotics to EMT6 tumor cells. *Proc. Am. Assoc. Cancer Res.*, **27**, 233.

ROCKWELL, S. (1983). Effects of mitomycin C alone and in combination with X-rays on EMT6 mouse mammary tumors *in vivo*. *J. Natl Cancer Inst.*, **71**, 765–771.

SIEGEL, D., BEALL, H., SENEKOWITSCH, C., KASAI, M., ARAI, H., GIBSON, N.W. & ROSS, D. (1992). Bioreductive activation of mitomycin C by DT-diaphorase. *Biochemistry*, **31**, 7879–7885.

SINGH, S.V., XU, B.H., MAURYA, A.K. & MIAN, A.M. (1992). Modulation of mitomycin C resistance by glutathione transferase inhibitor ethacrynic acid. *Biochim. Biophys. Acta*, **1137**, 257–263.

TOMASZ, M., LIPMAN, R., CHOWDARY, D., PAWLAK, J., VERDINE, G.L. & NAKANISHI, K. (1987). Isolation and structure of a covalent cross-link adduct between mitomycin C and DNA. *Science*, **235**, 1204–1208.

WILLSON, J.K.V., LONG, B.H., CHAKRABARTY, S., BRATTAIN, D.E. & BRATTAIN, M.G. (1985). Effects of BMY 25282, a mitomycin C analogue, in mitomycin C-resistant human colon cancer cells. *Cancer Res.*, **45**, 5281–5286.

WILLSON, J.K.V., CHAKRABARTY, S., LONG, B.H. & BRATTAIN, M.G. (1987). Deficient activation of mitomycin C in naive resistant human colon cancer cells. *Proc. Am. Assoc. Cancer Res.*, **23**, 286.

XU, B.H. & SINGH, S.V. (1992*a*). Effect of buthionine sulfoximine and ethacrynic acid on cytotoxic activity of mitomycin C analogues BMY 25282 and BMY 25067. *Cancer Res.*, **52**, 6666–6670.

XU, B.H. & SINGH, S.V. (1992*b*). Potentiation of mitomycin C cytotoxicity by glutathione depletion in a multi-drug resistant mouse leukemia cell line. *Cancer Lett.*, **66**, 49–53.

XU, B.H., GUPTA, V. & SINGH, S.V. (1993). Mechanism of resistance to mitomycin C (MMC) in a human bladder cancer cell line. *Proc. Am. Assoc. Cancer Res.*, **34**, 340.


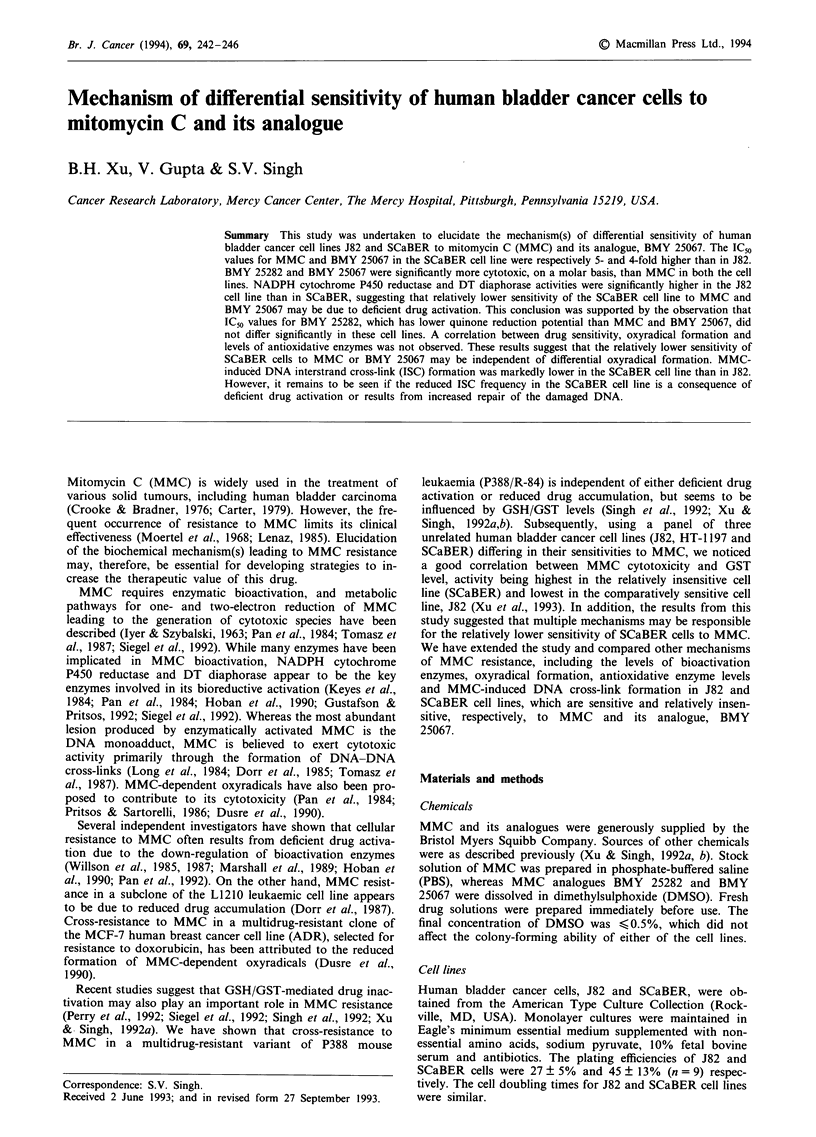

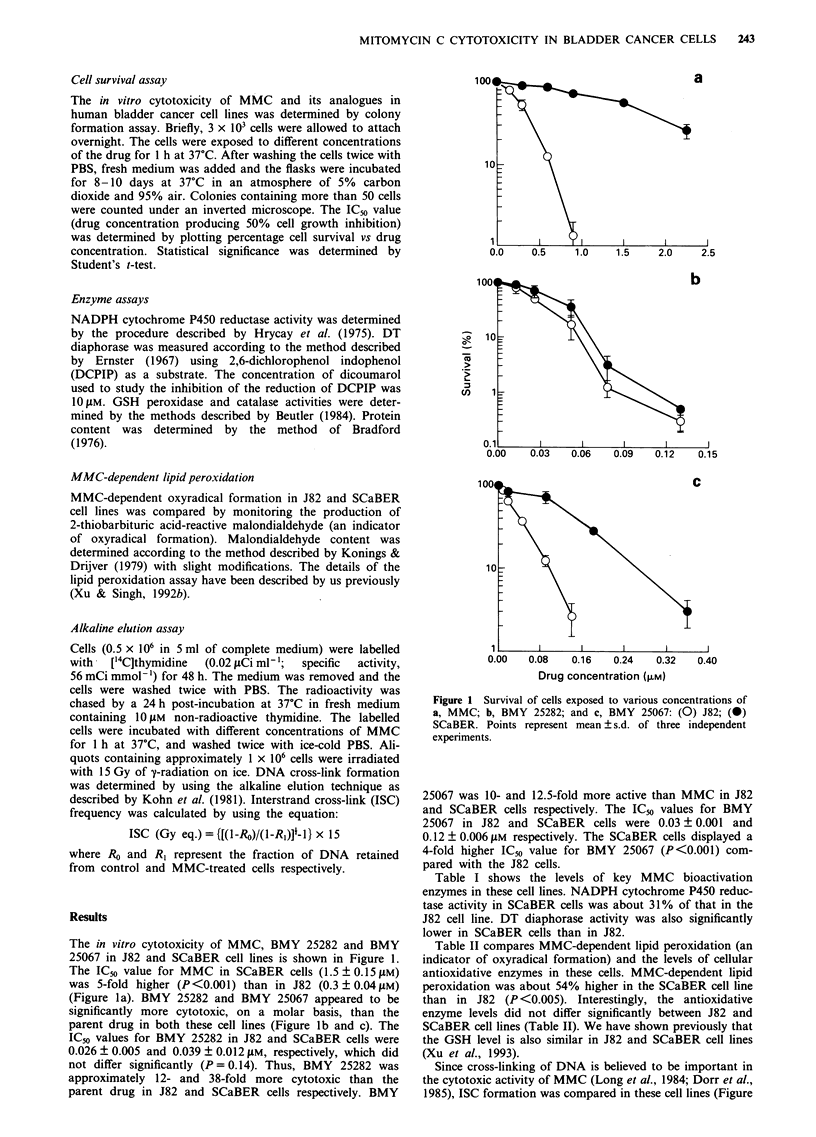

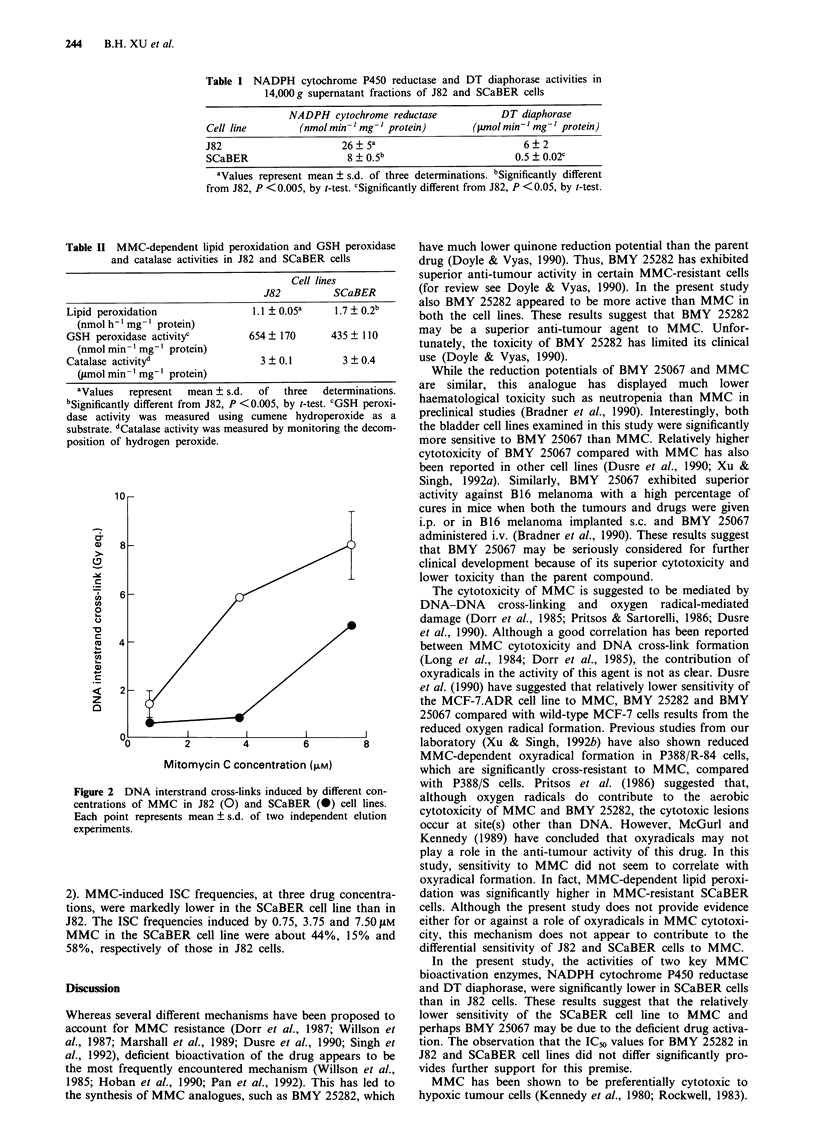

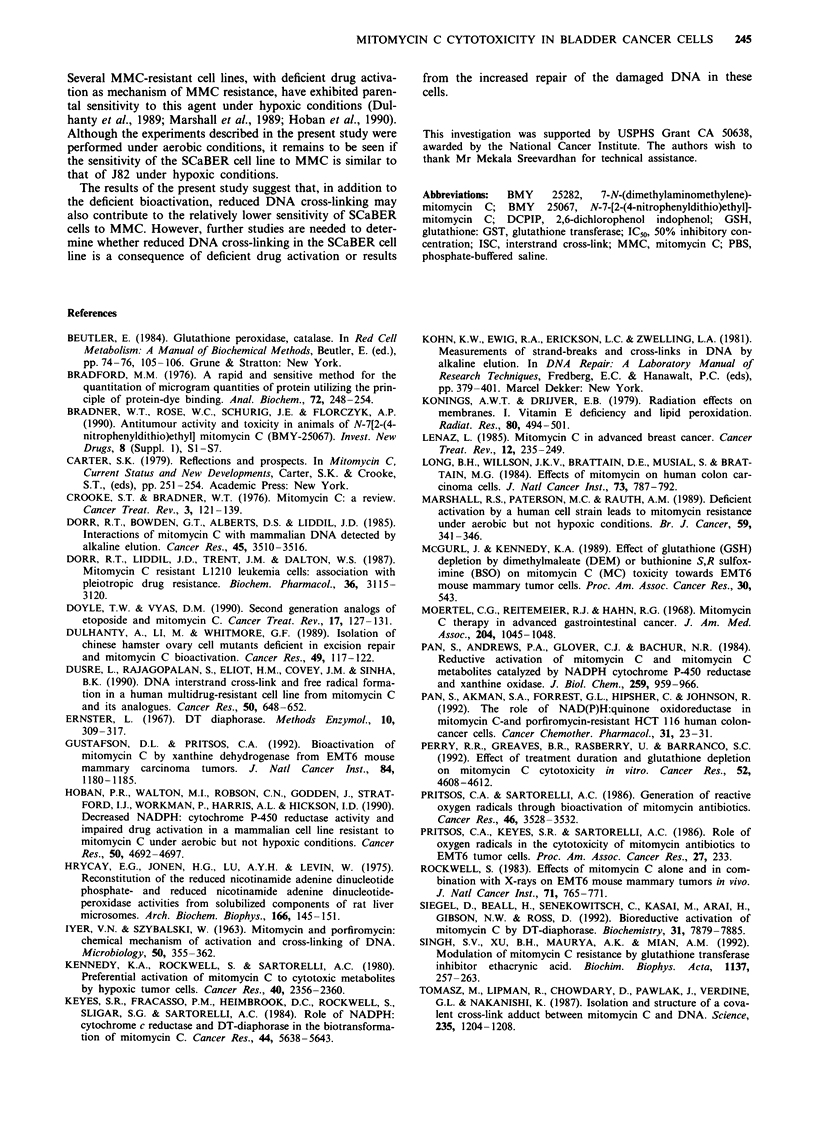

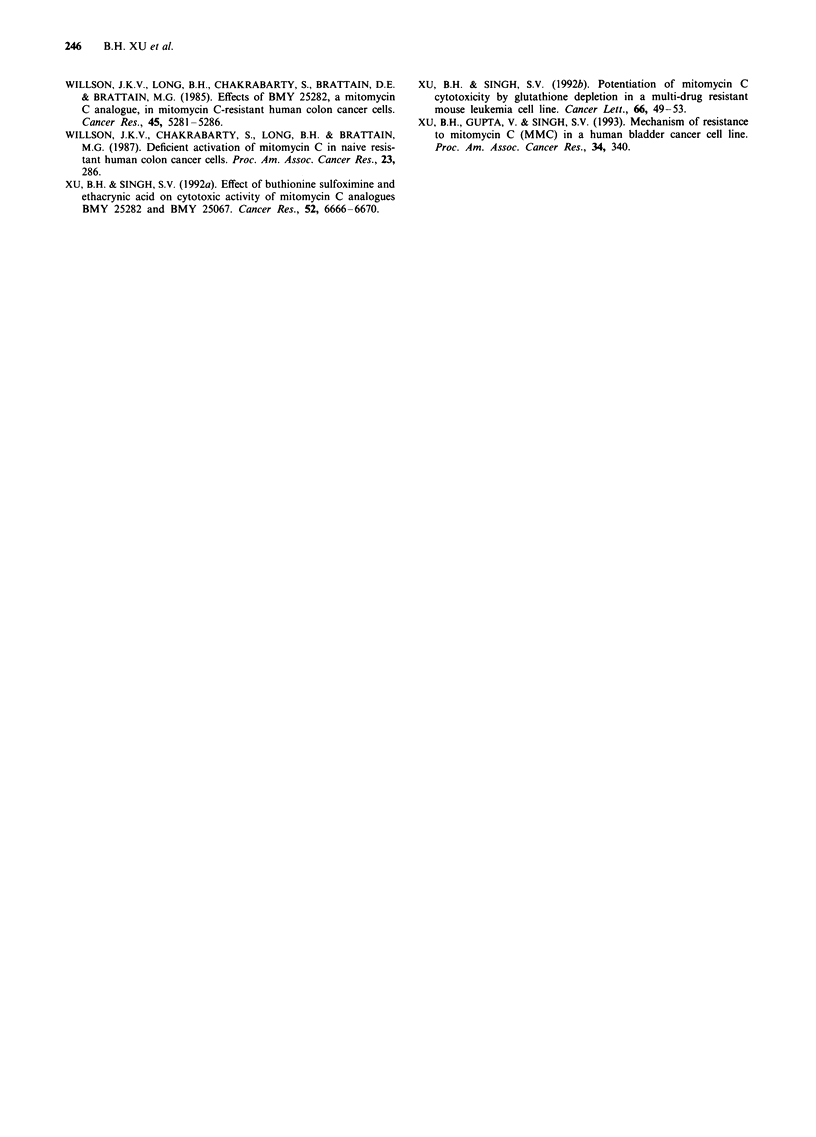


## References

[OCR_00455] Bradford M. M. (1976). A rapid and sensitive method for the quantitation of microgram quantities of protein utilizing the principle of protein-dye binding.. Anal Biochem.

[OCR_00460] Bradner W. T., Rose W. C., Schurig J. E., Florczyk A. P. (1990). Antitumor activity and toxicity in animals of N-7[2-(4-nitrophenyldithio) ethyl] mitomycin C (BMY-25067).. Invest New Drugs.

[OCR_00471] Crooke S. T., Bradner W. T. (1976). Mitomycin C: a review.. Cancer Treat Rev.

[OCR_00475] Dorr R. T., Bowden G. T., Alberts D. S., Liddil J. D. (1985). Interactions of mitomycin C with mammalian DNA detected by alkaline elution.. Cancer Res.

[OCR_00480] Dorr R. T., Liddil J. D., Trent J. M., Dalton W. S. (1987). Mitomycin C resistant L1210 leukemia cells: association with pleiotropic drug resistance.. Biochem Pharmacol.

[OCR_00486] Doyle T. W., Vyas D. M. (1990). Second generation analogs of etoposide and mitomycin C.. Cancer Treat Rev.

[OCR_00490] Dulhanty A. M., Li M., Whitmore G. F. (1989). Isolation of Chinese hamster ovary cell mutants deficient in excision repair and mitomycin C bioactivation.. Cancer Res.

[OCR_00495] Dusre L., Rajagopalan S., Eliot H. M., Covey J. M., Sinha B. K. (1990). DNA interstrand cross-link and free radical formation in a human multidrug-resistant cell line from mitomycin C and its analogues.. Cancer Res.

[OCR_00505] Gustafson D. L., Pritsos C. A. (1992). Bioactivation of mitomycin C by xanthine dehydrogenase from EMT6 mouse mammary carcinoma tumors.. J Natl Cancer Inst.

[OCR_00513] Hoban P. R., Walton M. I., Robson C. N., Godden J., Stratford I. J., Workman P., Harris A. L., Hickson I. D. (1990). Decreased NADPH:cytochrome P-450 reductase activity and impaired drug activation in a mammalian cell line resistant to mitomycin C under aerobic but not hypoxic conditions.. Cancer Res.

[OCR_00519] Hrycay E. G., Jonen H. G., Levin W., Lu A. Y. (1975). Reconstitution of the reduced nicotinamide adenine dinucleotide phosphate- and reduced nicotinamide adenine dinucleotide-peroxidase activities from solubilized components of rat liver microsomes.. Arch Biochem Biophys.

[OCR_00531] Kennedy K. A., Rockwell S., Sartorelli A. C. (1980). Preferential activation of mitomycin C to cytotoxic metabolites by hypoxic tumor cells.. Cancer Res.

[OCR_00536] Keyes S. R., Fracasso P. M., Heimbrook D. C., Rockwell S., Sligar S. G., Sartorelli A. C. (1984). Role of NADPH:cytochrome c reductase and DT-diaphorase in the biotransformation of mitomycin C1.. Cancer Res.

[OCR_00549] Konings A. W., Drijver E. B. (1979). Radiation effects on membranes. I. Vitamin E deficiency and lipid peroxidation.. Radiat Res.

[OCR_00554] Lenaz L. (1985). Mitomycin C in advanced breast cancer.. Cancer Treat Rev.

[OCR_00560] Long B. H., Willson J. K., Brattain D. E., Musial S., Brattain M. G. (1984). Effects of mitomycin on human colon carcinoma cells.. J Natl Cancer Inst.

[OCR_00563] Marshall R. S., Paterson M. C., Rauth A. M. (1989). Deficient activation by a human cell strain leads to mitomycin resistance under aerobic but not hypoxic conditions.. Br J Cancer.

[OCR_00576] Moertel C. G., Reitemeier R. J., Hahn R. G. (1968). Mitomycin C therapy in advanced gastrointestinal cancer.. JAMA.

[OCR_00587] Pan S. S., Akman S. A., Forrest G. L., Hipsher C., Johnson R. (1992). The role of NAD(P)H:quinone oxidoreductase in mitomycin C- and porfiromycin-resistant HCT 116 human colon-cancer cells.. Cancer Chemother Pharmacol.

[OCR_00581] Pan S. S., Andrews P. A., Glover C. J., Bachur N. R. (1984). Reductive activation of mitomycin C and mitomycin C metabolites catalyzed by NADPH-cytochrome P-450 reductase and xanthine oxidase.. J Biol Chem.

[OCR_00593] Perry R. R., Greaves B. R., Rasberry U., Barranco S. C. (1992). Effect of treatment duration and glutathione depletion on mitomycin C cytotoxicity in vitro.. Cancer Res.

[OCR_00599] Pritsos C. A., Sartorelli A. C. (1986). Generation of reactive oxygen radicals through bioactivation of mitomycin antibiotics.. Cancer Res.

[OCR_00609] Rockwell S. (1983). Effects of mitomycin C alone and in combination with X-rays on EMT6 mouse mammary tumors in vivo.. J Natl Cancer Inst.

[OCR_00614] Siegel D., Beall H., Senekowitsch C., Kasai M., Arai H., Gibson N. W., Ross D. (1992). Bioreductive activation of mitomycin C by DT-diaphorase.. Biochemistry.

[OCR_00619] Singh S. V., Xu B. H., Maurya A. K., Mian A. M. (1992). Modulation of mitomycin C resistance by glutathione transferase inhibitor ethacrynic acid.. Biochim Biophys Acta.

[OCR_00625] Tomasz M., Lipman R., Chowdary D., Pawlak J., Verdine G. L., Nakanishi K. (1987). Isolation and structure of a covalent cross-link adduct between mitomycin C and DNA.. Science.

[OCR_00633] Willson J. K., Long B. H., Chakrabarty S., Brattain D. E., Brattain M. G. (1985). Effects of BMY 25282, a mitomycin C analogue, in mitomycin C-resistant human colon cancer cells.. Cancer Res.

[OCR_00645] Xu B. H., Singh S. V. (1992). Effect of buthionine sulfoximine and ethacrynic acid on cytotoxic activity of mitomycin C analogues BMY 25282 and BMY 25067.. Cancer Res.

[OCR_00650] Xu B. H., Singh S. V. (1992). Potentiation of mitomycin C cytotoxicity by glutathione depletion in a multi-drug resistant mouse leukemia cell line.. Cancer Lett.

